# Could the estrobolome have a role in endometriosis pathogenesis and infertility? A systematic review

**DOI:** 10.1186/s12905-025-04195-z

**Published:** 2025-12-18

**Authors:** Stefania Saponara, Francesco Scicchitano, Maurizio Nicola D’Alterio, Caterina Chilà, Angelos Daniilidis, Salvatore Giovanni Vitale, Stefano Angioni

**Affiliations:** 1 Division of Gynecology and Obstetrics, Department of Surgical Sciences, University of Cagliari, Cagliari, Italy; 2 Department of Obstetrics and Gynecology, 2nd University Clinic of Obstetrics and Gynecology, Aristotele University of Thessaloniki, Thessaloniki, Greece; 3 Obstetrics and Gynecology Unit, “Gaspare Rodolico” University Hospital, Department of General Surgery and Medical Surgical Specialties, University of Catania, Catania, Italy

**Keywords:** Estrobolome, Gut microbiome, Immune system, β-glucuronidase, Endometriosis, Infertility, Dysbiosis

## Abstract

**Background:**

Endometriosis is a chronic, estrogen-dependent condition affecting 10% of reproductive-aged women, often associated with infertility and chronic pelvic pain. Recent evidence suggests that gut microbiota dysbiosis and alterations in the estrobolome, defined as the collection of bacterial genes involved in estrogen metabolism, may play a role in the pathogenesis of endometriosis and infertility.

**Methods:**

This systematic review was registered with PROSPERO (ID: CRD42024627464). A comprehensive search was conducted across PubMed, Embase, Scopus, Web of Science, Cochrane CENTRAL, ClinicalTrials.gov, and grey literature sources up to December 2024, without a lower date limit. The search included terms such as “estrobolome,” “endometriosis,” “infertility,” and “estrogen metabolism”. Original articles and clinical trials investigating the role of the estrobolome in endometriosis pathogenesis and infertility were included. Abstract-only studies, reviews, and non-English articles were excluded.

**Results:**

Five studies were analyzed, highlighting gut dysbiosis, estrobolome alterations, and immunological factors in endometriosis and infertile patients. Some investigations reported dysregulated or increased β-glucuronidase activity, suggesting a potential link between microbial estrogen metabolism and disease pathophysiology. These microbial and enzymatic alterations were accompanied by elevated inflammatory cytokines and persistent activation of immune cells, possibly contributing to local and systemic estrogen stimulation and lesion progression.

**Conclusions:**

Our analysis emphasizes how disruptions in estrogen-metabolizing bacterial pathways may contribute to the inflammatory and hormonal features observed in endometriosis and infertility. Given the associations observed, future studies should explore whether modulating the microbiota or estrogen metabolism can improve clinical outcomes in patients with endometriosis or infertility.

**Supplementary Information:**

The online version contains supplementary material available at 10.1186/s12905-025-04195-z.

## Background

Endometriosis is a chronic, estrogen-dependent gynecological condition with a multifactorial pathogenesis and an inflammatory nature. It is characterized by endometrial-like tissue outside the uterine cavity [1] and in distant organs, such as the lungs, liver, brain, and skin [2]. Affecting approximately 10% of the general female population, the prevalence of endometriosis rises significantly to 20–50% in women experiencing infertility or chronic pelvic pain [1, 3]. While symptoms investigation, new classification systems and treatment options are continuously improving in the whole gynecologic field [4–8], diagnosing and managing the endometriosis and/or infertile patient remains challenging, in medical, surgical and economical terms [9–13]. The complexity of this disease extends beyond traditional etiopathogenetic theories, encompassing a combination of genetic predispositions, immune dysregulation, hormonal imbalances, environmental influences, and alterations in the gut microbiome and metabolome [14–18].

Recently, increasing attention has been given to the role of the microbiome in gynecological conditions. Dysbiosis and estrobolome impairments are under investigation for their potential impact on hormone-dependent diseases such as endometriosis, cancers, and infertility [18–21]. The human microbiota, comprising diverse symbiotic microorganisms including viruses, bacteria, archaea, fungi and protozoa [22, 23], plays an important role in maintaining health by influencing epithelial gut function, the immune system, metabolism, and endocrine balance.

In a state of *eubiosis*, a healthy gut microbiota, producing a wide repertoire of enzymes and metabolites [24] supports a robust epithelial barrier in the gut, regulates inflammation, and maintains metabolic and endocrine stability [19, 24]. However, numerous factors, including hormonal fluctuations and dietary patterns, can disrupt microbial equilibrium, potentially triggering dysbiosis in predisposed individuals [25]. Characterized by the reduction of microbial diversity and imbalances in specific taxa, *dysbiosis* has been implicated in a wide range of non-gynecological diseases, including inflammatory bowel disease, diabetes, obesity, autoimmune disorders, cardiovascular disease and cancer [21, 26–28]. Several recent studies have proposed a link between microbial imbalance and the development of endometriosis and infertility [18, 29, 30] possibly by affecting the estrobolome [19, 20, 24, 28, 31].

The term estrobolome, introduced in 2011 by Plottel et al. [32], refers to the subset of bacterial genes involved in estrogen metabolism. The estrobolome produces enzymes such as β-glucuronidase, β-glucosidase, and β-galactosidase, which deconjugate estrogen metabolites (but also toxins, drugs and other substances), allowing them to re-enter circulation in their active forms [31, 33, 34]. Dysbiosis can lead to an overrepresentation of bacterial taxa enriched with the *gusB* gene, which encodes gut microbial β-glucuronidases (*gm*GUSB), the key functional component of the estrobolome [33, 35]. Altered estrobolome activity may increase estrogen bioavailability, contributing to the hyperestrogenic environment typical of endometriosis, infertility and other estrogen-dependent diseases [20, 21, 28, 36, 37].

Beyond hyperestrogenism, some authors report that microbial imbalances may exacerbate the inflammatory environment characteristic of endometriosis [38–44]. This occurs by disrupting the gut epithelial barrier, promoting bacterial translocation and reducing the production of critical protective metabolites such as short-chain fatty acids (SCFAs), bile acids, and tryptophan derivatives [38–44]. Typically, immune clearance mechanisms prevent ectopic implantation of endometrial cells. When this clearance fails, the lesions expand due to inflammation triggered by macrophages releasing pro-inflammatory cytokines and growth factors into the peritoneal cavity [45]. In endometriosis, the chronic activation of the immune system perpetuates a self-sustaining cycle of damage and repair. Immune cells, including neutrophils, Natural Killer cells (NK), B and T lymphocytes and macrophages, invade the endometrium, myometrium and peritoneum, enhancing cytokine production such as interleukins IL-1β, IL-6, Transforming Growth Factor-β (TGFβ) and other factors, like Hypoxia Induced Factor (HIF-1α). Combined with oxidative stress and insufficient iron scavenging in peritoneal fluid, these elements create a sustained pro-inflammatory environment that prompts lesions progression [46–52].

This evidence suggests that gut microbial imbalance and immune disruption might jointly influence estrogen-related pathways, offering a possible explanation for disease progression.

The aim of this systematic review is to critically evaluate the role of the estrobolome in the pathogenesis of endometriosis and infertility, by analyzing human and animal studies that investigate the relationship between microbial dysbiosis, estrogen metabolism, and immune system activation.

## Methods

### Search strategy

A systematic literature review was conducted to evaluate the impact of estrobolome modifications on the pathogenesis of endometriosis and infertility. The review protocol was registered with the International Prospective Register of Systematic Reviews (PROSPERO ID: CRD42024627464). Strictly following the Preferred Reporting Items for Systematic Reviews and Meta-Analyses (PRISMA) guidelines [53], we comprehensively searched the PubMed, EMBASE, CLINICALTRIALS.GOV, Scopus, Web of Science, and Cochrane CENTRAL databases. To ensure broader coverage, we also screened grey literature repositories (Zenodo, ProQuest, OpenGrey).

The search strategy combined controlled vocabulary terms (MeSH and Emtree) and free-text keywords related to the estrobolome, estrogen metabolism, gut microbiota, dysbiosis, and reproductive disorders, using Boolean operators to ensure sensitivity and specificity. Synonyms and variations of these terms (e.g., “β-glucuronidase,” “intestinal flora,” “female infertility,” and “subfertility”) were also included. The complete and reproducible search strings for each database are provided in Additional File 1. The search was limited to full-text, English-language articles available up to December 2024, with no lower date restriction applied. To ensure that no studies of relevance were omitted, the bibliographies of all articles retrieved were manually cross-referenced.

Two reviewers (FS and CC) conducted the literature screening independently and data extraction was carried out collaboratively by the same authors. Extracted data included study characteristics, the samples and analysis methods employed, the type of technologies used, and the outcomes of each study. Any disagreements regarding study inclusion were resolved via consultative discussions with two additional authors (MND and SGV). We included the present study designs: case-control studies, retrospective and prospective studies, randomized and non-randomized controlled trials and multicenter studies. Narrative and systematic reviews, abstracts-only publications, non-English language studies, opinion articles, editorials, case reports, case series, correspondence, and commentaries were excluded. No corresponding authors were contacted to obtain missing data or clarifications, as all included studies provided sufficient information for data extraction and qualitative synthesis.

### Risk of bias

Two reviewers (SS and AD) independently assessed the methodological quality of the human studies included in this review using the Newcastle-Ottawa Scale (NOS) [54]. The quality of the studies was evaluated across three key domains: “Selection” (including representativeness of the cohort and ascertainment of exposure), “Comparability” (control for confounding factors), and “Outcome” (adequacy of follow-up and assessment of outcomes). Each domain was scored based on predefined criteria, with a maximum score of 9 points. Studies scoring 7–9 points were categorized as having a low risk of bias, while those scoring 5–6 points were considered moderate risk, and those scoring ≤ 4 points were deemed at high risk of bias. Any reviewer assessment discrepancies were resolved through discussion and consensus with a third reviewer (MND). SYRCLE’s Risk of Bias Tool was applied for animal studies to evaluate the methodological quality, focusing on ten domains. Random sequence generation, allocation concealment, blinding of caregivers and investigators, blinding of outcome assessment, incomplete outcome data, selective outcome reporting, baseline characteristics, animal housing conditions, contamination, and other sources of bias [55]. Each domain was categorized as low risk, unclear risk, or high risk of bias based on predefined criteria. Discrepancies between reviewers were resolved by discussion and, if necessary, by consulting a third reviewer (MND).

## Results

### Study selection and included studies

**Fig. 1:**
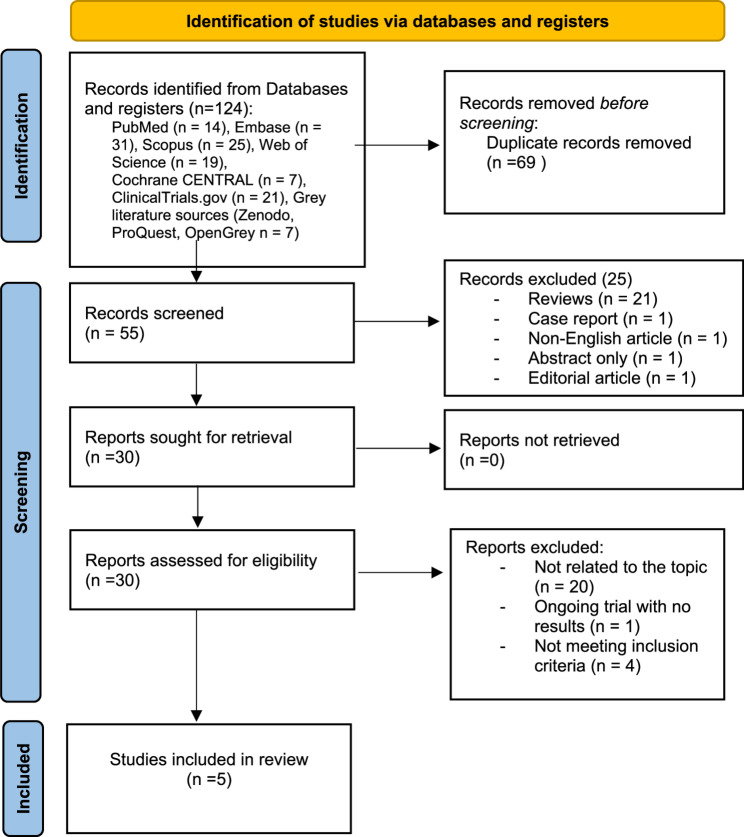
Flow diagram of the selection process

In our systematic review, we initially identified 124 records across all databases and registries. After removing 69 duplicate records, 55 records were screened based on title and abstract. During this initial screening phase, 25 records were excluded because they did not meet the inclusion criteria. Specifically, the excluded items comprised review articles (*n* = 21), one case report, one non-English publication, one abstract-only record, and one editorial article. Subsequently, 30 full-text reports were retrieved and assessed for eligibility. All full texts were successfully obtained for detailed evaluation. Following this assessment, 25 studies were excluded: 20 were not directly related to the research question, one was an ongoing clinical trial without available results, and four did not satisfy the predefined inclusion criteria. At the end of the selection process, five eligible case-control studies were included. Among these, three studies focused exclusively on human patients [56–58], one on mice [48], and one included both human and mouse subjects [59]. All five studies investigated the relationship between endometriosis, dysbiosis, and microbiome changes [48, 56–59]; two studies explored endometriosis and the immune system, both using animal models [48, 59]; one examined infertility, dysbiosis, and the immune system [58]; and two studies assessed estrogen metabolites or estrogen receptor (ER) activity [57, 58]. The sample types varied across the studies. Stool samples were analyzed in all five studies [48, 56–59], while histological analyses of endometrial or other tissue samples were conducted in two studies [58, 59]. Additionally, urine samples were analyzed in one study [57] and peripheral blood samples were included in two studies [48, 59]. Only one study [56] conducted analyses adjusted for potential confounders (age, BMI, antibiotic use, and hormonal status). The other studies did not specify any confounder adjustment. Given the heterogeneity of study designs and outcomes, results were synthesized narratively rather than statistically. The study selection process is illustrated in Fig. 1, and a detailed summary of the included studies is presented in Table 1.

**Table 1:** Summary of the included studies

Author	Study type	Type of population	Case/Controls	Sample(s)	Outcome(s)	Result(s)
Prieto et al., 2024	Case-control	Human (*n* = 1000)	ENDO (*n* = 136)/controls (*n* = 864)	Stools	1. Microbiome changes 2. β-glucuronidase changes	1. No difference in α- and β-diversity ↓ Paraprevotella clara ↓ Parabacteroides sp. D26 2. No differences in β-glucuronidase and β-galactosidases activity/expression
Scarfò et al., 2024	Case-control	Human (*n* = 40)	Patients with primary infertility and RIF (all without endometriosis) *Cases*: Dysbiosis group (Lb < 90%) *Controls*: Eubiosis group (Lb < 90%)	Endometrial biopsy	1. Microbioma changes 2. β-glucuronidase changes 3. ERα/β activity 4. Cytokines	1. N/A α- and β-diversity ↓ Lactobacillus spp 2. ↑ β-glucuronidase activity 3. ↑ in ERβ activity 4. ↑ in IL-1β, HIF-1α No difference in ERα and IL-8
Pai et al., 2023	Case-control	Human (*n* = 51)	ENDO (*n* = 27)/controls (*n* = 24)	Stools Urines	1. Microbioma changes 2. β-glucuronidase 3. E2 metabolites variations	1. No difference in α- and β-diversity ↑ Erysipelotrichia class, ↑ Einsenbergella genus, ↑ Hungatella genus (↑ F/B ratio - *p* = 0.43) 2. No differences β-glucuronidase and β-glucosidase activity on fecal samples 3. ↑ estriol (*p* = 0.011) in stool ↑ 16-epiestriol (*p* = 0.018) ↑ 16α-hydroxyestrone (*p* = 0.016) ↑ 2-methoxyestradiol (*p* = 0.035) No changes in metabolites in urines
Wei et al., 2023	Case-control	Human and experimental mouse model	Human: Stool samples - ENDO (*n* = 28), Controls (*n* = 25) Peripheral blood: -ENDO (*n* = 35), Controls (*n* = 30) Histological samples: -EMs lesions (*n* = 100), NonEMs samples (*n* = 50), Normal endometrium (*n* = 50)	Human: - Stools - Peripheral blood - EMs lesion samples	Human: 1. Microbiome changes 2. β-glucuronidase changes 3. Immune system changes	Human: 1. No difference in α- and β-diversity ↑ Desulfobacterota (phylum) ↑ Desulfovibrionia (class) ↑ Desulfovibrionales (order) ↑ Desulfovibrionaceae (family) ↑ Desulfovibrio/Biophila (genus) ↑ Eggerthellaceae (family) ↑ Eubacteriales (order) ↑ Eubacteriaceae (family) ↑ Eubacterium (genus) 2. ↑ β-glucuronidase expression and E2 in EMs lesions ↑ β-glucuronidase levels under E2 stimulation 3. ↑migration and invasion by M2 polarized macrophage under β-glucuronidase and E2 stimulation
			Experimental Mice treated with E2 and β-glucuronidase injected in the tail every 3 days: - 0 mg/mouse (controls) - 20 mg, 100 mg β-glucuronidase + E2 (cases)	Mice: - Stools - Peripheral blood - Peritoneal fluid, vaginal lavage, endometrial smears	Mice: 1. Microbiome changes 2. β-glucuronidase changes 3. Immune system changes	Mice: 1. ↓ α-diversity in gut microbiome, no difference in β-diversity 2. ↑ β-glucuronidase in uterus and endometrial stromal cells in Ems 3. ↑ LPS under GUSB stimulation, ↑ macrophage polarization to M2 over M1 by β-glucuronidase ↑ Endometriotic lesions size/number
Alghatea et al., 2023	Case-control	Animal (BALB/c mouse model)	Cases: - OVx + END (ovariectomized, DES, endometrial transplant) Controls: - Naïve (no treatment) - Naïve + ENDO (recipient, endometrial transplant) - OVx + VEH (donor, DES + ovariectomy)	- Stools - Peripheral Blood - Peritoneal Fluid - Uterine Fluid - Tissues	1. Microbiome Changes 2. Immune system changes 3. Metabolite variations 4. Cell respiration	1. N/A α- and β-diversity ↑ Ruminococcus spp ↑ Tenericutes (Phylum) ↑ Mollicutes (Class) ↑ Anaeroplasmatales (Order) ↑ Anaeroplasma (Genus) ↑ Lachnospiraceae (Family) ↑ Coprococcus (Genus) 2. ↑ Inflammatory cells in endometrium, myometrium, peritoneum ↑ NK and T cells ↑ WBC (neutrophils 3. ↓ SCFA 4. ↑ Mitochondrial ATP production in immune cells

*Abbreviations:ENDO* Endometriosis, *EMs* Endometriotic lesions, *RIF* Recurrent Implantation Failure, *Lb* Lactobacillus, *ERα* Estrogen Receptor Alpha, *ERβ* Estrogen Receptor Beta, *IL-1β* Interleukin-1 beta, *IL-8* Interleukin-8, *HIF-1α* Hypoxia-Inducible Factor 1-alpha, *E2* Estradiol, *F/B ratio* Firmicutes/Bacteroidetes ratio, *OVx* Ovariectomized, *DES* Diethylstilbestrol, *VEH* Vehicle, *NK* Natural Killer (cells), *WBC* White Blood Cells, *SCFA* Short-Chain Fatty Acids, *LPS* Lipopolysaccharide, *GUSB* Gut microbial β-glucuronidase, *N/A* Not Assessed

### Risk of bias assessment

#### Human studies 

The NOS assessment revealed scores between 6 and 8, indicating a moderate to low risk of bias (Additional File 2). Most studies showed strong cohort representativeness and appropriate exposure ascertainment. However, weaknesses were observed in controlling for confounders, with only Scarfò et al. [58] adequately addressing this aspect. Follow-up completeness was another limitation, affecting overall scores in some cases.

#### Animal studies

The SYRCLE’s Risk of Bias Tool evaluation highlighted a moderate risk of bias for both studies [48, 59] (Additional File 3). Strengths included low risk in handling incomplete data and selective reporting. However, critical weaknesses were identified in randomization, allocation concealment, and blinding, which were often unclear or insufficiently detailed.

### Estrobolome and dysbiosis in endometriosis

Four of the five eligible studies investigated the consequences of dysbiosis on the alteration of the estrobolome [48, 56, 57, 59].

Prieto and colleagues explored the gut microbiome in a large metagenomic investigation encompassing 1000 women (136 women with endometriosis and 864 healthy controls) [56]. Interestingly, their 16 S rRNA gene analysis on fresh stool samples found no significant differences in gut microbiome composition, function, or diversity (both α and β) between the two groups (*p* > 0.05). They identified 28 bacterial species with differential abundance, with *Clostridium* sp. CAG:307 (*p* = 0.019) and *Acinetobacter* sp. CAG:196 (*p* = 0.017) enriched in the endometriosis group, and *Ruminococcus* sp. CAG:177 (*p* = 0.023) and *Roseburia* sp. CAG:45 (*p* = 0.005) decreased compared to controls. Moreover, they detected a decrease in *Paraprevotella Clara* and *Parabacteroides* sp. D26 in women with endometriosis but noteworthy, none of these differences remained significant after False Discovery Rate (FDR) correction. Regarding the estrobolome, Prieto et al. analyzed 156 estrogen-related enzyme pathways, including β-glucuronidases and β-galactosidases, but observed no significant differences between the two groups (*p* > 0.05) [56].

Using stool and urine samples, Pai et al. [57] analyzed gut microbial composition and enzymatic activity variations in a cohort of 51 Taiwanese women (27 with endometriosis and 24 controls). Their analysis, including 16 S rRNA sequencing and estrogen metabolite profiling via LC/T-MS, showed no significant differences in α- or β-diversity (richness, evenness, or composition). In contrast, the gut microbiota of women with endometriosis exhibited a certain level of dysbiosis in terms of enrichment of some bacterial taxa including *the Erysipelotrichia class* (*p* = 0.0286), *Erysipelotrichales order* (*p* = 0.0286), *Erysipelotrichaceae famil*y (*p* = 0.0286), *Eisenbergiella genus* (*p* = 0.0474), and *Hungatella genus* (*p* = 0.0497). Secondary testing with Welch’s test confirmed higher levels of Erysipelotrichia (*p* = 0.036 – at the class level), Erysipelotrichales (*p* = 0.036), and Micrococcales (*p* = 0.039) in the endometriosis group. Although not statistically significant, a higher Firmicutes/Bacteroidetes (F/B) ratio was observed in endometriosis patients (0.81 vs. 0.73, *p* = 0.4269). In addition, neither the control nor the endometriosis group was significantly enriched with aerobic or anaerobic bacteria. Regarding the enzymatic evaluation, Pai and colleagues showed no significant differences in either β-glucuronidase levels (1823.45 U/L in controls vs. 1480.09 U/L in endometriosis, *p* = 0.35) or β-glucosidase activity. However, significantly elevated levels of estrogen metabolites, including estriol (*p* = 0.011), 16α-hydroxyestrone (*p* = 0.016), and 2-methoxyestradiol (*p* = 0.035), were detected in the endometriosis group.

In 2023, Wei and colleagues [59] conducted a case-control study to evaluate the impact of gut dysbiosis on estrobolome in patients with endometriosis (EMs) and mouse models. Their research analyzed gut dysbiosis, β-glucuronidase expression in EMs (including under E2 stimulation), macrophage polarization, cytokine secretion, and its effects on endometrial stromal cells (ESCs). They also investigated the impact of β-glucuronidase injections at varying concentrations in mice.

In the human investigation, they enrolled women aged 18 to 45 with a histological diagnosis of endometriosis. Sample collection involved gathering peripheral blood (35 EMs samples and 30 control samples), stools (28 EMs samples and 25 control samples were suitable for analysis) and endometriosis lesions specimens. Analyses were performed using ELISA, immunohistochemistry (IHC), and immunofluorescence. The stool sample analysis revealed no significant differences in microbial α- or β-diversity between the EMs and control groups. However, specific microbial taxa, including Desulfobacterota phylum, Eubacteriales (order), Eubacteriaceae (family), Eubacterium (genus), and Eggerthellaceae (family) were significantly enriched in the EMs group. These findings suggest that endometriosis is associated with notable shifts in gut microbiome composition.

Regarding the interaction with estrobolome, β-glucuronidase and estradiol (E2) serum levels were significantly elevated in EMs patients compared to controls (β-glucuronidase: 0.46 ± 0.29 pg/mL vs. 0.19 ± 0.12 pg/mL). IHC analysis further showed increased β-glucuronidase expression in bowel and uterosacral ligament lesions compared to normal endometrial tissue.

In the mouse model, experimental endometriosis was induced in 6–8-week-old female C57BL/6 mice via intraperitoneal injection of endometrial tissue and subcutaneous administration of E2. Mice were divided into three groups based on the dose of β-glucuronidase injected into the tail vein: 0 mg (control group, injected with PBS), 20 mg, and 100 mg. The results showed that β-glucuronidase expression was higher in uterine stromal cells of EMs mice compared to controls, and its administration led to a dose-dependent increase in the volume and number of endometriotic lesions.

In another work in 2023, Alghetaa et al. used 6–8-week-old female BALB/c mice to investigate the effects of dysbiosis on experimental endometriosis. Endometriosis was induced using estrogen stimulation and intraperitoneal transplantation of endometrial tissue.

Mice were divided into two primary groups: naïve mice (N; untreated controls) and ovariectomized (OVX) mice. OVX mice received subcutaneous injections of 100 µg/kg of diethylstilbestrol (DES, a synthetic estrogen) on days 0 and 5. On day 7, OVX mice were subdivided into two groups: one group served as donors of endometrial tissue (OVX-VEH), and the other as recipients (OVX-END), receiving peritoneal injections of transplanted endometrial tissue. OVX-VEH mice were treated with PBS (placebo intraperitoneal injection of Phosphate Buffer Saline solution). Naïve mice were also subdivided into two groups: one received endometrial transplants (Naïve + END), while the other received no further treatment. On days 10, 15, and 20, the OVX-END and OVX-VEH groups continued receiving DES injections, while Naïve + END and naïve controls were left under the natural hormonal influence of intact ovaries. At the endpoint, mice were euthanized, and peritoneal fluid and lesion samples were analyzed.

The data indicated that Endometrial transplantation led to the emergence of distinct bacterial populations in each treatment group. In all groups except naïve controls, endometrial transplantation enriched β-glucuronidase-producing bacteria like *Ruminococcus* spp, which are associated with estrogen metabolism and are commonly found within the Firmicutes phylum.

### Estrobolome, dysbiosis and immune system

Two of five studies investigated the relationship between dysbiosis and inflammation in endometriosis: one in experimental endometriosis mice models [48] and one both in humans and in mice [59].

As cited before, the study of Wei et al. [59] also examined the interplay between immune system dynamics and estrobolome changes in the development of endometriosis (EMs) in both human patients and mice [59]. In human patients, β-glucuronidase and E2 levels were elevated in the serum of the EMs group, with β-glucuronidase levels particularly high in endometriosis lesions. β-glucuronidase was shown to promote enhanced Endometrial Stomal Cells (ESC) invasion and migration in a wound-healing assay. Moreover, M2 macrophage polarization was significantly higher than M1 polarization in EMs lesions, especially in bowel lesions.

In vitro immunohistochemical (IHC) analysis using THP-1 cells (a cancer-derived monocyte-macrophage model) demonstrated that M2 macrophages produced three times more β-glucuronidase than M1 macrophages. Administration of E2 (10⁻⁷M) stimulated β-glucuronidase production in both M2 and M1 macrophages, leading to elevated M2 marker expression and cytokine secretion. Furthermore, ESC co-cultured with β-glucuronidase-treated M0 macrophage supernatants showed higher optical density measurements, indicating increased proliferation compared to placebo treatment.

In the mouse model, α-diversity was lower in EMs mice compared to controls. Additionally, increasing doses of β-glucuronidase led to higher lipopolysaccharide (LPS) levels in the peritoneal fluid, signaling inflammation, and contributed to an increase in the size and number of endometriotic lesions.

Regarding inflammation, Alghetaa and colleagues [48] argued that there was a correlation between dysbiosis and immuno-metabolic system dysregulation in mice with endometriosis. Locally, histopathological analysis showed increased inflammatory cell infiltration, predominantly neutrophils, in the endometrium and myometrium of the OVX + END group, leading to an endo-myometritis-like condition.

Systemically, OVX + END mice exhibited elevated levels of inflammatory cells, including Natural Killer (NK) cells, CD3 + T cells, and CD3 + NK + cells in the peritoneal cavity, along with higher total white blood cell (WBC) counts. This was accompanied by a decreased lymphocyte count across all groups (except the naïve group) but with a significantly higher percentage of neutrophils in the peripheral blood of both Naïve + END and OVX + END mice compared to controls. Additionally, a significant increase (*p* < 0.05) in the proliferative capacity of inflammatory cells was documented in the OVX + END and Naïve + END groups.

### Estrobolome, dysbiosis and infertility

Only one study investigated the correlation between estrobolome imbalance, endometrial dysbiosis and infertility [58]. In 2024, Scarfò and colleagues investigated inflammatory markers in infertile women with endometrial dysbiosis compared to those with eubiosis, focusing on patients without endometriosis [58]. All participants experienced primary infertility for at least three years, with ≥ 3 failed embryo transfers despite good-quality embryos. The participants, aged between 35 and 41 years, underwent in-vitro fertilization (IVF) treatment and endometrial sampling.

The study found that patients with endometrial dysbiosis exhibited significantly higher levels of the pro-inflammatory cytokine IL-1β (*p* < 0.0001) and hypoxia-inducible factor-1α (HIF-1α; *p* = 0.0053). IL-1β levels rose from 27.5 pg/mL in eubiotic patients to 70.4 pg/mL in dysbiotic patients, reflecting a pronounced inflammatory state associated with dysbiosis. HIF-1α levels also showed a significant increase, with dysbiotic patients recording 22.9 ng/µg compared to 11.9 ng/µg in patients with eubiosis.

While pro-inflammatory markers IL-1β and HIF-1α were significantly elevated, no notable differences were observed in IL-6, IL-8, or IL-10 levels between the groups. This suggests that the inflammatory profile in dysbiotic patients may selectively affect specific pathways rather than triggering a generalized cytokine response.

Regarding β-glucuronidase activity and estrogen receptor beta (ERβ) expression, both were significantly elevated in the dysbiosis group (*p* < 0.0001 and *p* = 0.0044, respectively). Notably, Lactobacilli abundance was inversely correlated with β-glucuronidase activity and ERβ expression. This finding indicates that alterations in estrogen-metabolizing enzymes could influence the expression of estrogen receptors. Conversely, no significant changes were observed in ERα expression (*p* = 0.3514).

## Discussion

### Main findings

This systematic review examined how dysbiosis-related estrobolome alterations may initiate or contribute to the progression of endometriosis and influence infertility.

The included studies primarily addressed changes in microbial composition, specifically diversity and taxa abundance, alongside β-glucuronidase activity as a marker of estrobolome function. Moreover, they explored the immune system’s response to these alterations, focusing on the involvement of immune cell populations and pro-inflammatory cytokines.

The evidence on microbial diversity in endometriosis remains inconclusive. While three reviewed studies [56, 57, 59] found no significant differences in microbial α- and β-diversity between endometriosis and control groups, the other studies [48, 58] did not assess this parameter. Despite the lack of consensus, some studies reported an enrichment in specific bacterial taxa such as Firmicutes (*Desulfobacterota*, *Eubacteriales*, *Erysipelotrichia*) and *Ruminococcus* spp., a known β-glucuronidase-producing genus [19, 33, 60]. However, variability in β-glucuronidase gene distribution among these taxa complicates the interpretation of whether dysbiosis universally impacts the estrobolome.

In the human gut microbiome, two distinct bacterial β-glucuronidase-encoding genes, *gus* and BG, have been identified [35, 61]. Metagenomic datasets showed that *gus* genes were predominantly associated with *Firmicutes*, while BG genes were shared between *Firmicutes* and *Bacteroidetes* [62, 63]. This variability in β-glucuronidase expression across bacterial taxa suggests that enriched taxa may contribute to estrobolome activity in different ways, or not at al. Furthermore, this variability in enzyme expression among taxa likely underpins the inconsistencies observed across studies. Enrichment of bacterial taxa lacking glucuronidase-encoding genes or possessing different variants (*gus* versus BG) can result in significant differences in findings. Additionally, differences between human and animal microbiomes make it challenging to extrapolate findings from animal models of endometriosis, which do not naturally develop the disease [64, 65].

Only one study directly evaluated the *Firmicutes*/*Bacteroidetes* ratio [57], finding a higher ratio in the endometriosis group, even with no statistical significance (0.81 vs. 0.73, *p* = 0.4269). In literature, the relationship between the Firmicutes/Bacteroidetes (F/B) ratio and host health remains debated as obesity and endometriosis are more often linked to an increased F/B ratio and a lesser microbial diversity [66], whilst lean women with healthy gut microbiota have lower [67].

Contradictory findings, such as higher F/B ratios in endometriosis-induced mice [68] and lower ratios in other studies [69] underscore the complexity of these associations and their potential link to β-glucuronidase activity, suggesting potential alterations in the estrobolome and estrogen metabolism in endometriosis but further research is needed to elucidate the estrobolome’s role in this condition.

The correlation between gut dysbiosis, inflammation, and hormones has been widely proven [24, 33, 59, 70–73]. The gut microbiome in endometriosis may be enriched with β-glucuronidase-producing bacteria, which may lead to a hyperestrogenic state and exacerbate the condition. In our research, results are slightly discordant. Some studies [58, 59] observed elevated β-glucuronidase levels in endometriosis and infertile patients, indicating that endometriosis and infertility may be linked independently by inflammation and β-glucuronidase enrichment leading to hyperestrogenism. Conversely, no variation in β-glucuronidase levels, either in stools or in urines, has been described by Pai and his colleagues [57]. However, they identified significantly increased levels of estriol and three estrogen metabolites in stool samples, alongside enrichment of specific *Firmicutes* taxa (*Erysipelotrichia* class, *Einsebergella* genus, and *Hungatella* genus), many of which are known to be β-glucuronidase producer and potentially associated with hyperestrogenism. This discrepancy may be explained or depends on the multitude of post-transcriptional events that may regulate an enzyme production, the different β-glucuronidase isoforms produced by different niche microbiota or bacterial taxa, as reported in the literature [24, 56, 57]. Moreover, it is worth noting that the study of Pai and colleagues [57] received the lowest score on the Newcastle-Ottawa Scale (NOS) for human studies, which may reflect potential biases or methodological limitations contributing to the observed discrepancies (Table 1).

Gut dysbiosis also disrupts the gut epithelial barrier, leading to increased bacterial transepithelial translocation and, as a reaction, to systemic inflammation. This process is exacerbated by the endotoxemia induced by lipopolysaccharide S (LPS) and pro-inflammatory cytokines, such as interleukins IL-1β and IL-17α [44, 45, 74, 75]. In endometriosis, this inflammatory cascade is further influenced by sex hormones, which regulate immune cells, including macrophages, NK cells, T and B lymphocytes, and neutrophils. These immune cells play dual roles: they are essential for normal endometrial cyclic function but also contribute to the chronic inflammation characteristic of endometriosis [51, 76–82]. During the perimenstrual phase, immune activation facilitates the controlled degradation of tissue. Macrophages, for instance, scavenge apoptotic endometrial cells while promoting tissue repair through wound-healing mechanisms. This transition from pro-inflammatory to an anti-inflammatory environment is critical for tissue homeostasis [83]. However, in endometriosis, this regulation is impaired [49]. Macrophages fail to efficiently clear retrograde menstrual blood, cellular debris, and iron deposits in the peritoneum, leading to a persistent pro-inflammatory immune profile in both eutopic and ectopic endometrial cells. Estrogen can exacerbate this dysfunction by inducing macrophage polarization from the anti-inflammatory M1 phenotype to a pro-inflammatory M2 phenotype, which sustains chronic inflammation and hinders immune surveillance on ectopic endometrial cells [77, 84]. This data aligns with the results described by Wei [59] and Alghetaa [48] who reported increased M2 macrophage infiltration and proliferation in endometriotic lesions. Notably, Wei and colleagues [59] reported a threefold increase in *β*-glucuronidase synthesis in M2 macrophages lysosomes under estrogen (E2) stimulation, along with elevated serum levels of *β*-glucuronidase and E2, highlighting the hormonal stimulus as a key driver of this immune dysregulation. At the molecular level, chronic stimulation of estrogen receptors (ERs) contributes to the formation of inflammasomes, which drive the production of pro-inflammatory cytokines, such as IL-1β. This signaling cascade enhances TGFβ-mediated adhesion of cells, promotes anti-apoptotic mechanisms, and facilitates the proliferation of endometrial stromal cells (ESCs), ultimately fueling neo-angiogenesis and lesion expansion [81–84]. These molecular pathways are consistent with the immune and hormonal dysfunctions observed in human and animal endometriosis studies.

B and T cells also play a critical role in this inflammatory microenvironment. IL-17, a key cytokine, links these two immune cell populations by stimulating B cells to produce autoantibodies and enhancing Th2 cell proliferation. This creates a feedback loop that perpetuates chronic inflammation, angiogenesis, and impaired immune surveillance of ectopic lesions [45, 85, 86].

Although less studied, neutrophils have been implicated in chronic inflammation within the endometriosis microenvironment. Elevated levels of chemotactic factors such as IL-8 recruit neutrophils to endometriotic lesions, where they exacerbate their inflammatory response [87, 88]. Estrogens also appear to influence neutrophil function and accumulation, but their precise role in endometriosis pathogenesis remains under investigation [80, 81, 89, 90]. Interestingly, immune dysfunction in endometriosis may also interfere with endometrial receptivity and implantation processes [1]. In a healthy uterus, NK cells help prepare the endometrium for the embryo. After implantation, they switch to an anti-inflammatory and tolerating state to help maintain a pregnancy [91, 92]. In endometriosis, disrupted estrogen signaling reduces NK cell functionality, impairing embryo implantation and pregnancy success. This disruption may be linked to ERβ stimulation, which suppresses NK cell maturation and decreases NK cell levels during implantation [51]. These findings align with the observation that 20–50% of infertile women have underlying endometriosis [1], with repeated implantation failure being a common complication [82, 89].

A similar inflammatory pattern has been observed by Scarfò et al. in the only study identified in our review that examined the relationship among dysbiosis, the estrobolome, and infertility [58]. They reported increased β-glucuronidase activity, enhanced ERβ activity, and elevated levels of IL-1β and HIF-1α in infertile women with dysbiosis (defined as *Lactobacillus spp* < 90% in endometrial samples). Interestingly, the reduction in *Lactobacillus spp* (which has much variability in *β*-glucuronidase production among different species) was inversely correlated with β-glucuronidase activity and ERβ expression, suggesting a direct link between dysbiosis and hormonal dysregulation. However, the study did not identify the bacterial taxa responsible for the increased enzyme activity, introducing potential analytical biases and leaving the exact mechanisms unclear.

**Fig. 2:**
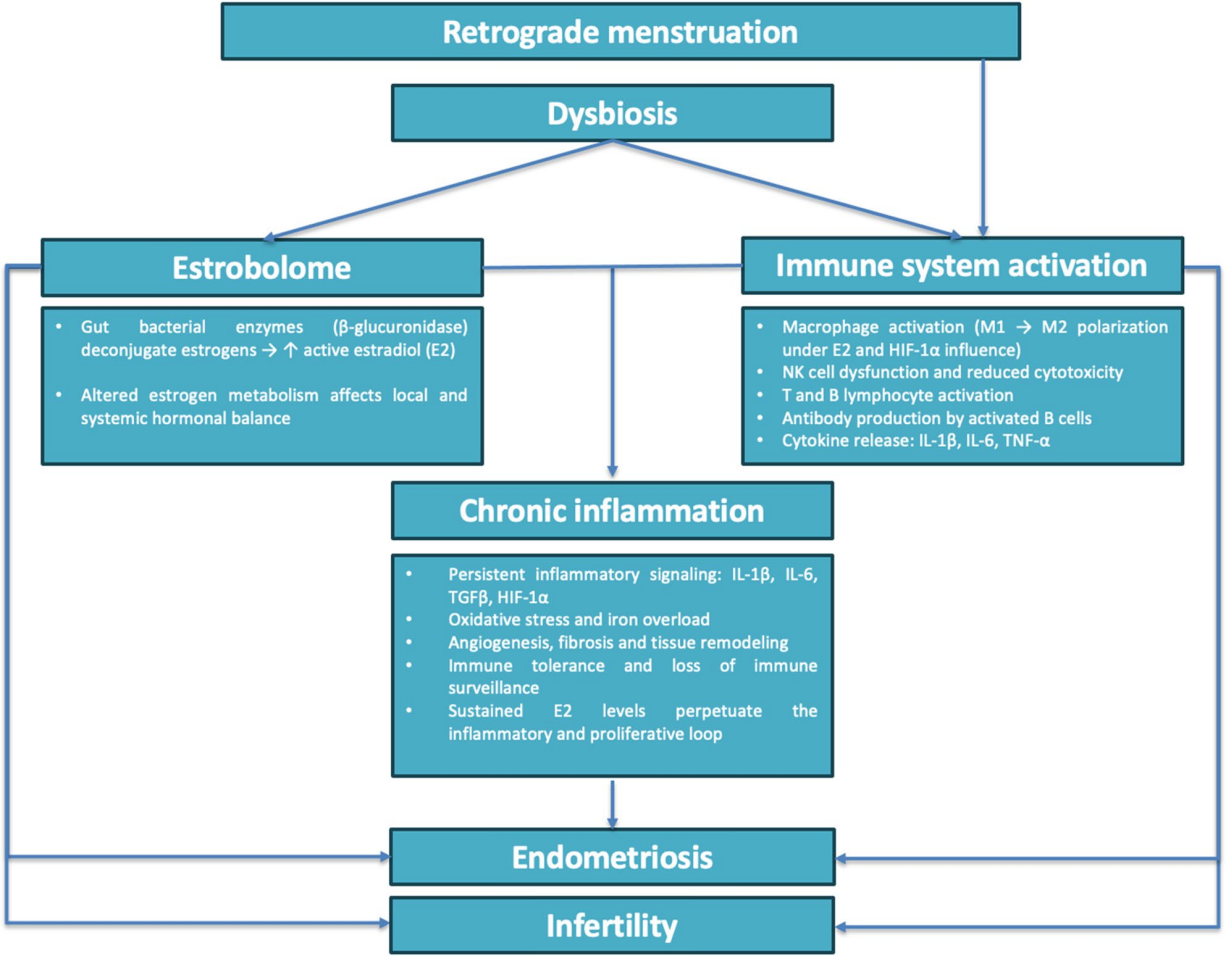
Putative connections between dysbiosis, estrobolome, immune system, endometriosis and infertility

Figure 2 provides a schematic overview of the interactions between gut dysbiosis, estrobolome activity, immune dysregulation, and hormonal imbalance, illustrating how these interconnected systems may contribute to the development of endometriosis and associated infertility.

Although only five studies met our inclusion criteria by directly assessing the estrobolome in the context of endometriosis or infertility, numerous other investigations have explored the broader interplay between gut dysbiosis, immune activation, and inflammatory pathways in endometriosis [18, 25, 27, 29, 44–47]. These studies, which were not included in our systematic synthesis because they did not specifically evaluate estrobolome-related activity, consistently report alterations in microbial diversity, impaired intestinal barrier function, and enhanced production of pro-inflammatory cytokines such as IL-1β, IL-6, and TNF-α [31, 34, 35, 44–47]. Such findings support the hypothesis that dysbiosis may act as an upstream trigger of systemic and peritoneal inflammation, contributing to lesion progression and immune dysfunction.

Nevertheless, based on the limited dataset analyzed here, the association between dysbiosis, estrobolome activity, and inflammatory or immune alterations should be regarded as hypothesis-generating rather than confirmatory. Future integrative studies combining metagenomic, metabolomic, and immunologic approaches are warranted to elucidate the causal mechanisms linking microbial estrogen metabolism to the inflammatory microenvironment characteristic of endometriosis.

### Strengths and limitations

To our knowledge, this is the first systematic review that specifically addresses how estrobolome alterations may influence endometriosis and infertility, providing valuable insights into the intricate mechanisms linking gut microbiota, estrogen metabolism, and systemic inflammation. While the studies included provide crucial perspectives, significant gaps remain. Considerable heterogeneity exists among the included studies, particularly regarding microbiota assessment methods and samples types, including both human and animal models. Additionally, most of the included studies are observational, with no randomized controlled trials (RCTs) identified, limiting the ability to establish causal relationships. Methodological weaknesses, such as inconsistent confounder control in human studies and lack of randomization in animal studies, underline the need for greater rigor in future research. Furthermore, only one study directly evaluates the impact of the estrobolome on fertility, underscoring the need for more robust research in this area to draw definitive conclusions. Finally, most included studies did not adjust for major confounding factors, such as age, BMI, or hormonal exposure. The absence of statistical adjustment may have influenced the observed associations between microbiota composition, estrobolome activity, and endometriosis or infertility.

## Conclusions

The evidence reviewed in this study suggests that alterations in the gut microbiota and immune function may influence estrogen metabolism in patients with endometriosis and infertility. Although the precise mechanisms remain incompletely understood, the involvement of the estrobolome appears to be a recurring element across several studies. These alterations may contribute to a hormonal and inflammatory environment that favors lesion persistence and reduced reproductive competence.

Data from both human and animal models point to a link between dysbiosis and immune cell imbalances, including increased macrophage polarization and impaired NK cell activity, which may further impact endometrial receptivity and implantation.

Interestingly, the coexistence of endometriosis and infertility may not always reflect a direct causal relationship. Rather, both conditions could stem from a common set of underlying biological disturbances, including genetic, hormonal, immunological, and microbial factors. This broader view may help reframe how these conditions are studied and managed clinically.

However, the heterogeneity and limitations of the studies analyzed, including the lack of randomized controlled trials and standardization in microbiota analysis, emphasize the need for further high-quality research.

Despite these gaps, microbiota-targeted therapies, such as probiotics or enzyme inhibitors, may offer new opportunities to modulate inflammation and hormonal pathways. Further research is needed to clarify these mechanisms and develop tailored interventions.

## Supplementary Information

Supplementary Material 1.

Supplementary Material 2.

Supplementary Material 3.

Supplementary Material 4.

## Data Availability

Data sharing is not applicable to this article as no datasets were generated or analysed during the current study.

## Abbreviations

EMs: Endometriosis
ER: Estrogen Receptor
ERβ: Estrogen Receptor Beta
ERα: Estrogen Receptor Alpha
ESCs: Endometrial Stromal Cells
F/B ratio: Firmicutes/Bacteroidetes ratio
FDR: False Discovery Rate
HIF-1α: Hypoxia-Inducible Factor 1-alpha
IHC: Immunohistochemistry
IL: Interleukin
IVF: In Vitro Fertilization
LC/T-MS: Liquid Chromatography/Tandem Mass Spectrometry
LPS: Lipopolysaccharide
NK: Natural Killer
NOS: Newcastle-Ottawa Scale
OVX: Ovariectomized
PBS: Phosphate Buffered Saline
PRISMA: Preferred Reporting Items for Systematic Reviews and Meta-Analyses
SCFA: Short-Chain Fatty Acids
SYRCLE: Systematic Review Centre for Laboratory animal Experimentation
THP-1: Human Monocyte-Macrophage Cell Line
TGFβ: Transforming Growth Factor Beta
WBC: White Blood Cells
E2: Estradiol
DES: Diethylstilbestrol
Naïve + END: Naïve mice with endometrial transplant
gmGUSB: Gut microbial β-glucuronidase
GUS: β-glucuronidase gene
BG: β-glucuronidase gene

## References

[1] Zondervan KT, Becker CM, Missmer SA, Endometriosis. N Engl J Med. 2020;382:1244–56. 32212520 10.1056/NEJMra1810764

[2] Andres MP, Arcoverde FVL, Souza CCC, Fernandes LFC, Abrão MS, Kho RM. Extrapelvic endometriosis: a systematic review. J Minim Invasive Gynecol. 2020;27:373–89. 31618674 10.1016/j.jmig.2019.10.004

[3] Leone Roberti Maggiore U, Chiappa V, Ceccaroni M, Roviglione G, Savelli L, Ferrero S, et al. Epidemiology of infertility in women with endometriosis. Best Pract Res Clin Obstet Gynaecol. 2024. 10.1016/j.bpobgyn.2023.102454. 38183767 10.1016/j.bpobgyn.2023.102454

[4] D’Alterio MN, Nappi L, Vitale SG, Agus M, Fanni D, Malzoni M, et al. Evaluation of ovarian reserve and recurrence rate after DWLS diode laser ovarian endometrioma vaporization (OMAlaser): A Prospective, Single-Arm, Multicenter, clinical trial. J Minim Invasive Gynecol. 2025;32(3):279–87. 39477011 10.1016/j.jmig.2024.10.021

[5] D’Alterio MN, Scicchitano F, Fanni D, Faa G, Laganà AS, Noventa M, et al. Ex vivo myolysis with dual wavelengths diode laser system: macroscopic and histopathological examination. Clin Exp Obstet Gynecol. 2021;48:875–82.

[6] Vitale SG, Saponara S, Succu AG, Sicilia G, Martsidis K, D’Alterio MN, et al. Efficacy and safety of non-ablative dual wavelength diode laser therapy for genitourinary syndrome of menopause: a single-center prospective study. Adv Ther. 2024;41(12):4617–27. 39470875 10.1007/s12325-024-03004-7

[7] Keckstein J, Hudelist G. Classification of deep endometriosis (DE) including bowel endometriosis: from r-ASRM to #Enzian-classification. Best Pract Res Clin Obstet Gynaecol. 2021;71:27–37. 33558167 10.1016/j.bpobgyn.2020.11.004

[8] Urman B, Yakin K, Ertas S, Alper E, Aksakal E, Riemma G, et al. Fertility and anatomical outcomes following hysteroscopic adhesiolysis: an 11-year retrospective cohort study to validate a new classification system for intrauterine adhesions (Urman-Vitale Classification System). Int J Gynaecol Obstet. 2024;165:644–54. 38013507 10.1002/ijgo.15262

[9] D’Alterio MN, Saponara S, Dancona G, Russo M, Lagana AS, Sorrentino F, et al. Role of surgical treatment in endometriosis. Minerva Obstet Gynecol. 2021;73:317–32. 34008386 10.23736/S2724-606X.21.04737-7

[10] D’Alterio MN, Saponara S, Agus M, Laganà AS, Noventa M, Loi ES, et al. Medical and surgical interventions to improve the quality of life for endometriosis patients: a systematic review. Gynecol Surg. 2021;18:1–14.

[11] Angioni S, Saponara S, D’Ancona G, Sicilia G, D’Alterio MN, Vitale SG. Safety, efficacy, and cost-effectiveness of organ suspension in laparoscopic gynecologic surgery: a retrospective cohort study to validate an innovative technique: laparoscopic organ suspension sec. Angioni. Gynecol Obstet Invest. 2024;89(6):445–52. 38697034 10.1159/000538787

[12] Angioni S. New insights on endometriosis. Minerva Ginecol. 2017;69:438–9. 28545294 10.23736/S0026-4784.17.04089-8

[13] Vitale SG, Laganà AS, Török P, Lasmar RB, Carugno J, Palumbo M, et al. Virtual sonographic hysteroscopy in assisted reproduction: a retrospective cost-effectiveness analysis. Int J Gynaecol Obstet. 2022;156:112–8. 33615469 10.1002/ijgo.13651

[14] Deiana D, Gessa S, Anardu M, Daniilidis A, Nappi L, D’Alterio MN, et al. Genetics of endometriosis: a comprehensive review. Gynecol Endocrinol. 2019;35:553–8. 30909768 10.1080/09513590.2019.1588244

[15] Angioni S, D’alterio MN, Coiana A, Anni F, Gessa S, Deiana D. Genetic characterization of endometriosis patients: review of the literature and a prospective cohort study on a Mediterranean population. Int J Mol Sci. 2020;21(5):1765. 32143537 10.3390/ijms21051765 PMC7084255

[16] Angioni S, Saponara S, Vitale SG. Metabolomics analysis in endometriosis patients: is it a step toward the future? Gynecol Endocrinol. 2023;39(1):2227276. 37369250 10.1080/09513590.2023.2227276

[17] Angioni S, Saponara S, Succu AG, Sigilli M, Scicchitano F, D’Alterio MN. Metabolomic Characteristics in Endometriosis Patients.In: Genazzani, AR, Nisolle M, Petraglia F, Taylor RN, editors. Endometriosis Pathogenesis, Clinical Impact and Management. ISGE Series. Cham: Springer; 2021. p. 9–17. 10.1007/978-3-030-57866-4_2.

[18] D’Alterio MN, Giuliani C, Scicchitano F, Laganà AS, Oltolina NM, Sorrentino F, et al. Possible role of Microbiome in the pathogenesis of endometriosis. Minerva Obstet Gynecol. 2021;73:193–214. 33851803 10.23736/S2724-606X.21.04788-2

[19] Salliss ME, Farland LV, Mahnert ND, Herbst-Kralovetz MM. The role of gut and genital microbiota and the estrobolome in endometriosis, infertility and chronic pelvic pain. Hum Reprod Update. 2022;28:92–131. 10.1093/humupd/dmab035 34718567

[20] Jiang I, Yong PJ, Allaire C, Bedaiwy MA. Intricate connections between the microbiota and endometriosis. Int J Mol Sci. 2021. 10.3390/ijms22115644. 34073257 10.3390/ijms22115644 PMC8198999

[21] Borella F, Carosso AR, Cosma S, Preti M, Collemi G, Cassoni P, et al. Gut microbiota and gynecological cancers: a summary of pathogenetic mechanisms and future directions. ACS Infect Dis. 2021;7:987–1009. 33848139 10.1021/acsinfecdis.0c00839

[22] Kim Y, Koh IS, Rho M. Deciphering the human microbiome using next-generation sequencing data and bioinformatics approaches. Methods. 2015;79–80:52–9. 25448477 10.1016/j.ymeth.2014.10.022

[23] Marchesi JR, Ravel J. The vocabulary of microbiome research: a proposal. Microbiome. 2015. 10.1186/s40168-015-0094-5. 26229597 10.1186/s40168-015-0094-5 PMC4520061

[24] Baker JM, Al-Nakkash L, Herbst-Kralovetz MM. Estrogen–gut microbiome axis: physiological and clinical implications. Maturitas. 2017;103:45–53. 28778332 10.1016/j.maturitas.2017.06.025

[25] Rinninella E, Raoul P, Cintoni M, Franceschi F, Miggiano GAD, Gasbarrini A, et al. What is the healthy gut microbiota composition? A changing ecosystem across age, environment, diet, and diseases. Microorganisms. 2019;7(1):14. 30634578 10.3390/microorganisms7010014 PMC6351938

[26] Wang MY, Sang LX, Sun SY. Gut microbiota and female health. World J Gastroenterol. 2024;30:1655–62. 38617735 10.3748/wjg.v30.i12.1655 PMC11008377

[27] Belizário JE, Faintuch J. Microbiome and gut dysbiosis. Exp Suppl. 2018;109:459–76. 30535609 10.1007/978-3-319-74932-7_13

[28] Cocomazzi G, Del Pup L, Contu V, Maggio G, Parmegiani L, Ciampaglia W, et al. Gynecological cancers and microbiota dynamics: insights into pathogenesis and therapy. Int J Mol Sci. 2024. 10.3390/ijms25042237. 38396914 10.3390/ijms25042237 PMC10889201

[29] Sobstyl A, Chałupnik A, Mertowska P, Grywalska E. How do microorganisms influence the development of endometriosis? Participation of genital, intestinal and oral microbiota in metabolic regulation and immunopathogenesis of endometriosis. Int J Mol Sci. 2023. 10.3390/ijms241310920. 37446108 10.3390/ijms241310920 PMC10341671

[30] Laschke MW, Menger MD. The gut microbiota: a puppet master in the pathogenesis of endometriosis? Am J Obstet Gynecol. 2016;215:e681-4. 10.1016/j.ajog.2016.02.036 26901277

[31] Ervin SM, Li H, Lim L, Roberts LR, Liang X, Mani S, et al. Gut microbial β-glucuronidases reactivate estrogens as components of the estrobolome that reactivate estrogens. J Biol Chem. 2019;294:18586–99. 31636122 10.1074/jbc.RA119.010950 PMC6901331

[32] Plottel CS, Blaser MJ. Microbiome and malignancy. Cell Host Microbe. 2011;10:324–35. 22018233 10.1016/j.chom.2011.10.003 PMC3264051

[33] Hu S, Ding Q, Zhang W, Kang M, Ma J, Zhao L. Gut microbial beta-glucuronidase: a vital regulator in female estrogen metabolism. Gut Microbes. 2023. 10.1080/19490976.2023.2236749. 37559394 10.1080/19490976.2023.2236749 PMC10416750

[34] Ning L, Hong J. Gut microbial β-Glucuronidase: a key regulator of endobiotic homeostasis. Cell Host Microbe. 2024;32:783–5. 38870895 10.1016/j.chom.2024.05.007

[35] Dabek M, McCrae SI, Stevens VJ, Duncan SH, Louis P. Distribution of β-glucosidase and β-glucuronidase activity and of β-glucuronidase gene Gus in human colonic bacteria. FEMS Microbiol Ecol. 2008;66:487–95. 18537837 10.1111/j.1574-6941.2008.00520.x

[36] Sui Y, Wu J, Chen J. The role of gut microbial β-Glucuronidase in estrogen reactivation and breast cancer. Front Cell Dev Biol. 2021;9:631552. 34458248 10.3389/fcell.2021.631552 PMC8388929

[37] Zulli K, Bianco B, Mafra FA, Teles JS, Christofolini DM, Barbosa CP. Polymorphism of the estrogen receptor β gene is related to infertility and infertility-associated endometriosis. Arq Bras Endocrinol Metabol. 2010;54:567–71. 20857063 10.1590/s0004-27302010000600010

[38] Fattahi Y, Heidari HR, Khosroushahi AY. Review of short-chain fatty acids effects on the immune system and cancer. Food Biosci. 2020;38:100793.

[39] Amabebe E, Anumba DOC. Female gut and genital tract microbiota-induced crosstalk and differential effects of short-chain fatty acids on immune sequelae. Front Immunol. 2020;11:553047. 10.3389/fimmu.2020.02184 PMC7511578 33013918

[40] Zeng H, Umar S, Rust B, Lazarova D, Bordonaro M. Secondary bile acids and short chain fatty acids in the colon: A focus on colonic Microbiome, cell Proliferation, Inflammation, and cancer. Int J Mol Sci 2019. 2019;20:20:1214. 10.3390/ijms20051214 PMC6429521 30862015

[41] Yang H, Zhou H, Zhuang L, Auwerx J, Schoonjans K, Wang X, et al. Plasma membrane-bound G protein-coupled bile acid receptor attenuates liver ischemia/reperfusion injury via the Inhibition of toll-like receptor 4 signaling in mice. Liver Transpl. 2017;23:63–74. 27597295 10.1002/lt.24628

[42] Jia W, Xie G, Jia W. Bile acid–microbiota crosstalk in gastrointestinal inflammation and carcinogenesis. Nat Rev Gastroenterol Hepatol. 2017;15(2):111–28. 29018272 10.1038/nrgastro.2017.119 PMC5899973

[43] Liu M, Peng R, Tian C, Shi J, Ma J, Shi R, et al. Effects of the gut microbiota and its metabolite short-chain fatty acids on endometriosis. Front Cell Infect Microbiol. 2024. 10.3389/fcimb.2024.1373004. 38938880 10.3389/fcimb.2024.1373004 PMC11208329

[44] Deitch EA. The role of intestinal barrier failure and bacterial translocation in the development of systemic infection and multiple organ failure. Arch Surg. 1990;125:403–4. 2407230 10.1001/archsurg.1990.01410150125024

[45] Ahn SH, Edwards AK, Singh SS, Young SL, Lessey BA, Tayade C. IL-17A contributes to the pathogenesis of endometriosis by triggering proinflammatory cytokines and angiogenic growth factors. J Immunol. 2015;195:2591–600. 26259585 10.4049/jimmunol.1501138 PMC4561197

[46] Alghetaa HF, Mohammed A, Nagarkatti M, Nagarkatti P. Gut dysbiosis and immunological profile in endometriosis. J Immunol. 2018;200(1_Supplement):108.6-108.6.

[47] Belkaid Y, Hand TW. Role of the microbiota in immunity and inflammation. Cell. 2014;157:121–41. 24679531 10.1016/j.cell.2014.03.011 PMC4056765

[48] Alghetaa H, Mohammed A. Estrobolome dysregulation is associated with altered immunometabolism in a mouse model of endometriosis. Front Endocrinol (Lausanne). 2023;14:1261781. 38144564 10.3389/fendo.2023.1261781 PMC10748389

[49] Capobianco A, Rovere-Querini P. Endometriosis, a disease of the macrophage. Front Immunol. 2013;4:38169. 10.3389/fimmu.2013.00009 PMC3556586 23372570

[50] Yuan Y, Li Y, Zhao W, Hu Y, Zhou C, Long T, et al. WNT4 promotes macrophage polarization via granulosa cell M-CSF and reduces granulosa cell apoptosis in endometriosis. Cytokine. 2023;172:156400. 37839333 10.1016/j.cyto.2023.156400

[51] Yang S, Wang H, Li D, Li M. An estrogen-NK cells regulatory axis in endometriosis, related infertility, and miscarriage. Int J Mol Sci. 2024. 10.3390/ijms25063362. 38542336 10.3390/ijms25063362 PMC10970045

[52] Gou Y, Li X, Li P, Zhang H, Xu T, Wang H, et al. Estrogen receptor β upregulates CCL2 via NF-κB signaling in endometriotic stromal cells and recruits macrophages to promote the pathogenesis of endometriosis. Hum Reprod. 2019;34:646–58. 30838396 10.1093/humrep/dez019

[53] Page MJ, McKenzie JE, Bossuyt PM, Boutron I, Hoffmann TC, Mulrow CD, et al. The PRISMA 2020 statement: an updated guideline for reporting systematic reviews. BMJ. 2021;372:n71. 10.1136/bmj.n71. 10.1136/bmj.n71 PMC8005924 33782057

[54] Stang A. Critical evaluation of the Newcastle-Ottawa scale for the assessment of the quality of nonrandomized studies in meta-analyses. Eur J Epidemiol. 2010;25:603–5. 20652370 10.1007/s10654-010-9491-z

[55] Hooijmans CR, Rovers MM, De Vries RBM, Leenaars M, Ritskes-Hoitinga M, Langendam MW. SYRCLE’s risk of bias tool for animal studies. BMC Med Res Methodol. 2014;14:1–9. 24667063 10.1186/1471-2288-14-43 PMC4230647

[56] Pérez-Prieto I, Vargas E, Salas-Espejo E, Lüll K, Canha-Gouveia A, Pérez LA, et al. Gut microbiome in endometriosis: a cohort study on 1000 individuals. BMC Med. 2024. 10.1186/s12916-024-03503-y. 39020289 10.1186/s12916-024-03503-y PMC11256574

[57] Pai AHY, Wang YW, Lu PC, Wu HM, Xu JL, Huang HY. Gut microbiome-estrobolome profile in reproductive-age women with endometriosis. Int J Mol Sci. 2023. 10.3390/ijms242216301. 38003489 10.3390/ijms242216301 PMC10671785

[58] Scarfò G, Daniele S, Chelucci E, Papini F, Epifani F, Ruggiero M, et al. Endometrial dysbiosis: a possible association with estrobolome alteration. Biomolecules. 2024. 10.3390/biom14101325. 39456258 10.3390/biom14101325 PMC11506823

[59] Wei Y, Tan H, Yang R, Yang F, Liu D, Huang B, et al. Gut dysbiosis-derived β-glucuronidase promotes the development of endometriosis. Fertil Steril. 2023;120(3 Pt 2):682–94. 37178109 10.1016/j.fertnstert.2023.03.032

[60] Beaud D, Tailliez P, Aba-Mondoloni J. Genetic characterization of the β-glucuronidase enzyme from a human intestinal bacterium *Ruminococcus gnavus*. Microbiol (N Y). 2005;151:2323–30. 10.1099/mic.0.27712-0 16000722

[61] Gloux K, Berteau O, El Oumami H, Béguet F, Leclerc M, Doré J. A metagenomic β-glucuronidase uncovers a core adaptive function of the human intestinal Microbiome. Proc Natl Acad Sci U S A. 2011;108(SUPPL 1):4539–46. 20615998 10.1073/pnas.1000066107 PMC3063586

[62] Qin J, Li R, Raes J, Arumugam M, Burgdorf KS, Manichanh C, et al. A human gut microbial gene catalog established by metagenomic sequencing. Nature. 2010;464:59. 20203603 10.1038/nature08821 PMC3779803

[63] McIntosh FM, Maison N, Holtrop G, Young P, Stevens VJ, Ince J, et al. Phylogenetic distribution of genes encoding β-glucuronidase activity in human colonic bacteria and the impact of diet on faecal glycosidase activities. Environ Microbiol. 2012;14:1876–87. 22364273 10.1111/j.1462-2920.2012.02711.x

[64] Bellofiore N, Ellery SJ, Mamrot J, Walker DW, Temple-Smith P, Dickinson H. First evidence of a menstruating rodent: the spiny mouse (*Acomys cahirinus*). Am J Obstet Gynecol. 2017;216:e401-4011. 10.1016/j.ajog.2016.07.041 27503621

[65] Burns KA, Pearson AM, Slack JL, Por ED, Scribner AN, Eti NA, et al. Endometriosis in the mouse: challenges and progress toward a ‘best fit’ murine model. Front Physiol. 2022;12:806574. 35095566 10.3389/fphys.2021.806574 PMC8794744

[66] Magne F, Gotteland M, Gauthier L, Zazueta A, Pesoa S, Navarrete P, et al. The firmicutes/bacteroidetes ratio: a relevant marker of gut dysbiosis in obese patients? Nutrients. 2020. 10.3390/nu12051474. 32438689 10.3390/nu12051474 PMC7285218

[67] Turnbaugh PJ, Ley RE, Mahowald MA, Magrini V, Mardis ER, Gordon JI. An obesity-associated gut Microbiome with increased capacity for energy harvest. Nat 2006. 2006;444:7122. 10.1038/nature05414 17183312

[68] Yuan M, Li D, Zhang Z, Sun H, An M, Wang G. Endometriosis induces gut microbiota alterations in mice. Hum Reprod. 2018;33:607–16. 29462324 10.1093/humrep/dex372

[69] Chadchan SB, Cheng M, Parnell LA, Yin Y, Schriefer A, Mysorekar IU, et al. Antibiotic therapy with metronidazole reduces endometriosis disease progression in mice: a potential role for gut microbiota. Hum Reprod. 2019;34:1106–16. 31037294 10.1093/humrep/dez041 PMC6554192

[70] McKinnon BD, Bertschi D, Bersinger NA, Mueller MD. Inflammation and nerve fiber interaction in endometriotic pain. Trends Endocrinol Metab. 2015;26:1–10. 25465987 10.1016/j.tem.2014.10.003

[71] Seaman HE, Ballard KD, Wright JT, De Vries CS. Endometriosis and its coexistence with irritable bowel syndrome and pelvic inflammatory disease: findings from a National case–control study—Part 2. BJOG. 2008;115:1392–6. 18715239 10.1111/j.1471-0528.2008.01879.x

[72] Markle JGM, Frank DN, Mortin-Toth S, Robertson CE, Feazel LM, U Rolle-Kampczyk, et al. Sex differences in the gut Microbiome drive hormone-dependent regulation of autoimmunity. Sci (1979). 2013;339:1084–8. 10.1126/science.1233521 23328391

[73] Walsh J, Olavarria-Ramirez L, Lach G, Boehme M, Dinan TG, Cryan JF, et al. Impact of host and environmental factors on β-glucuronidase enzymatic activity: implications for Gastrointestinal serotonin. Am J Physiol Gastrointest Liver Physiol. 2020;318:G816–26. 32146834 10.1152/ajpgi.00026.2020

[74] Shreiner AB, Kao JY, Young VB. The gut microbiome in health and in disease. Curr Opin Gastroenterol. 2015;31:69–75. 25394236 10.1097/MOG.0000000000000139 PMC4290017

[75] Gazvani R, Templeton A. Peritoneal environment, cytokines and angiogenesis in the pathophysiology of endometriosis. Reproduction. 2002;123:217–26. 11866688 10.1530/rep.0.1230217

[76] Liang Y, Xie H, Wu J, Liu D, Yao S. Villainous role of estrogen in macrophage-nerve interaction in endometriosis. Reprod Biol Endocrinol. 2018;16:1–11. 30518376 10.1186/s12958-018-0441-z PMC6282253

[77] Jensen AL, Collins J, Shipman EP, Wira CR, Guyre PM, Pioli PA. A subset of human uterine endometrial macrophages is alternatively activated. Am J Reprod Immunol. 2012;68:374–86. 22882270 10.1111/j.1600-0897.2012.01181.x PMC3468696

[78] Wang Y, Chen H, Wang NL, Guo HY, Fu Y, Xue S, et al. Combined 17β-estradiol with TCDD promotes M2 polarization of macrophages in the endometriotic milieu with aid of the interaction between endometrial stromal cells and macrophages. PLoS One. 2015. 10.1371/journal.pone.0125559. 25950905 10.1371/journal.pone.0125559 PMC4423913

[79] Khan KN, Masuzaki H, Fujishita A, Kitajima M, Sekine I, Matsuyama T, et al. Estrogen and progesterone receptor expression in macrophages and regulation of hepatocyte growth factor by ovarian steroids in women with endometriosis. Hum Reprod. 2005;20:2004–13. 15831511 10.1093/humrep/deh897

[80] Milewski Ł, Dziunycz P, Barcz E, Radomski D, Roszkowski PI, Korczak-Kowalska G, et al. Increased levels of human neutrophil peptides 1, 2, and 3 in peritoneal fluid of patients with endometriosis: association with neutrophils, T cells and IL-8. J Reprod Immunol. 2011;91:64–70. 21831449 10.1016/j.jri.2011.05.008

[81] Symons LK, Miller JE, Tyryshkin K, Monsanto SP, Marks RM, Lingegowda H, et al. Neutrophil recruitment and function in endometriosis patients and a syngeneic murine model. FASEB J. 2020;34:1558–75. 31914688 10.1096/fj.201902272R

[82] Hannan NJ, Evans J, Salamonsen LA. Alternate roles for immune regulators: establishing endometrial receptivity for implantation. Expert Rev Clin Immunol. 2011;7:789–802. 22014020 10.1586/eci.11.65

[83] Henriet P, Gaide Chevronnay HP, Marbaix E. The endocrine and paracrine control of menstruation. Mol Cell Endocrinol. 2012;358:197–207. 21820486 10.1016/j.mce.2011.07.042

[84] Mei J, Zhou WJ, Li SY, Li MQ, Sun HX. Interleukin-22 secreted by ectopic endometrial stromal cells and natural killer cells promotes the recruitment of macrophages through promoting CCL2 secretion. Am J Reprod Immunol. 2019. 10.1111/aji.13166. 31295376 10.1111/aji.13166

[85] Symons LK, Miller JE, Kay VR, Marks RM, Liblik K, Koti M, et al. The immunopathophysiology of endometriosis. Trends Mol Med. 2018;24:748–62. 30054239 10.1016/j.molmed.2018.07.004

[86] Randall GW, Gantt PA, Poe-Zeigler RL, Bergmann CA, Noel ME, Strawbridge WR, et al. Serum antiendometrial antibodies and diagnosis of endometriosis. Am J Reprod Immunol. 2007;58:374–82. 17845208 10.1111/j.1600-0897.2007.00523.x

[87] Farland LV, Eliassen AH, Tamimi RM, Spiegelman D, Michels KB, Missmer SA. History of breast feeding and risk of incident endometriosis: prospective cohort study. BMJ. 2017;358:j3778. 10.1136/bmj.j3778. 10.1136/bmj.j3778 PMC5574033 28851765

[88] Singh N, Sethi A. Endometritis - diagnosis,treatment and its impact on fertility - a scoping review. JBRA Assist Reprod. 2022;26:538–46. 35621273 10.5935/1518-0557.20220015 PMC9355436

[89] Tariverdian N, Siedentopf F, Rücke M, Blois SM, Klapp BF, Kentenich H, et al. Intraperitoneal immune cell status in infertile women with and without endometriosis. J Reprod Immunol. 2009;80:80–90. 19375804 10.1016/j.jri.2008.12.005

[90] Chung HH, Or YZ, Shrestha S, Loh JT, Lim CL, Ong Z, et al. Estrogen reprograms the activity of neutrophils to foster protumoral microenvironment during mammary involution. Sci Rep. 2017;7:46485. 28429725 10.1038/srep46485 PMC5399373

[91] Zhang X, Wei H. Role of decidual natural killer cells in human pregnancy and related pregnancy complications. Front Immunol. 2021;12:728291. 34512661 10.3389/fimmu.2021.728291 PMC8426434

[92] Wasilewska A, Grabowska M, Moskalik-Kierat D, Brzoza M, Laudański P, Garley M. Immunological aspects of infertility—the role of KIR receptors and HLA-C antigen. Cells. 2023;13:59. 38201263 10.3390/cells13010059 PMC10778566

