# Supplementary material for: Could the estrobolome have a role in endometriosis pathogenesis and infertility? A systematic review
Source: BMC Womens Health. 2025 Dec 18;26:43. doi: 10.1186/s12905-025-04195-z (PMC12821278; doi:10.1186/s12905-025-04195-z)
Supplement: Supplementary file 1 — Supplementary Material 1. [file 12905_2025_4195_MOESM1_ESM.docx]

**Additional file 1**. Databases search strategies

| **Database** | **Search string** |
| --- | --- |
| **PubMed** | ((“estrobolome”[All Fields] OR “estrogen metabolism”[MeSH Terms] OR “estrogen metabolism”[All Fields] OR “oestrogen metabolism”[All Fields] OR “beta-glucuronidase”[All Fields] OR “beta glucuronidase”[All Fields]) AND (“gut microbiota”[MeSH Terms] OR “gut microbiota”[All Fields] OR “gut microbiome”[All Fields] OR “intestinal microbiota”[All Fields] OR “gut dysbiosis”[All Fields] OR “intestinal flora”[All Fields] OR “gut bacteria”[All Fields]) AND (“endometriosis”[MeSH Terms] OR “endometriosis”[All Fields] OR “female infertility”[MeSH Terms] OR “female infertility”[All Fields] OR “infertility”[MeSH Terms] OR “infertility”[All Fields] OR “subfertility”[All Fields])) |
| **Embase** | (‘estrobolome’/exp OR ‘estrobolome’:ab,ti OR ‘estrogen metabolism’/exp OR ‘estrogen metabolism’:ab,ti OR ‘oestrogen metabolism’:ab,ti OR ‘beta-glucuronidase’/exp OR ‘beta-glucuronidase’:ab,ti OR ‘beta glucuronidase’:ab,ti) AND (‘gut microbiota’/exp OR ‘gut microbiota’:ab,ti OR ‘gut microbiome’:ab,ti OR ‘intestinal microbiota’:ab,ti OR ‘gut dysbiosis’:ab,ti OR ‘intestinal flora’:ab,ti OR ‘gut bacteria’:ab,ti) AND (‘endometriosis’/exp OR ‘endometriosis’:ab,ti OR ‘female infertility’/exp OR ‘female infertility’:ab,ti OR ‘infertility’/exp OR ‘infertility’:ab,ti OR ‘subfertility’:ab,ti OR ‘reproductive disorder’:ab,ti) AND [<1966–2024]/py |
| **Scopus** | (TITLE-ABS-KEY (“estrobolome” OR “estrogen metabolism” OR “beta-glucuronidase”)) AND (TITLE-ABS-KEY (“gut microbiota” OR “gut microbiome” OR “intestinal microbiota” OR “gut dysbiosis”)) AND (TITLE-ABS-KEY (“endometriosis” OR “female infertility” OR “infertility”)) |
| **Web of Science** | (“estrobolome” OR “estrogen metabolism” OR “oestrogen metabolism” OR “beta-glucuronidase” OR “beta glucuronidase”) AND (“gut microbiota” OR “gut microbiome” OR “intestinal microbiota” OR “gut dysbiosis” OR “intestinal flora” OR “gut bacteria”) AND (“endometriosis” OR “female infertility” OR “infertility” OR “reproductive disorder” OR “subfertility”) |
| **Cochrane CENTRAL** | (“estrobolome” OR “estrogen metabolism” OR “beta-glucuronidase”) AND (“endometriosis” OR “infertility”) |
| **ClinicalTrials.gov** | (“endometriosis” OR “infertility”) AND (“gut microbiome” OR “estrobolome” OR “beta-glucuronidase”) |
| **Grey literature (OpenGrey, ProQuest, Zenodo, OpenAIRE)** | (“estrobolome” OR “estrogen metabolism” OR “beta-glucuronidase”) AND (“gut microbiota” OR “gut microbiome”) AND (“endometriosis” OR “infertility”) |
