# Supplementary material for: Could the estrobolome have a role in endometriosis pathogenesis and infertility? A systematic review
Source: BMC Womens Health. 2025 Dec 18;26:43. doi: 10.1186/s12905-025-04195-z (PMC12821278; doi:10.1186/s12905-025-04195-z)
Supplement: Supplementary file 2 — Supplementary Material 2. [file 12905_2025_4195_MOESM2_ESM.docx]

**Additional file 2.** Newcastle-Ottawa Scale (NOS) Evaluation for Human Studies

|  | **Selection** | | | | **Comparability** | **Outcome** | | |  |
| --- | --- | --- | --- | --- | --- | --- | --- | --- | --- |
|  | **Representativeness of exposed cohort** | **Selection of non-exposed cohort** | **Ascertainment of exposure** | **Outcome not present at the start of study** | **Control for confounding factors** | **Assessment of outcome** | **Follow-up long enough for outcomes to occur** | **Adequacy of follow-up** | **Total Score** |
| **Author** |  |  |  |  |  |  |  |  |  |
| Pai et al., 2023 | 1 | 1 | 1 | 0 | 1 | 1 | 1 | 0 | 6 |
| Prieto et al., 2024 | 1 | 1 | 1 | 1 | 1 | 1 | 1 | 0 | 7 |
| Scarfò et al., 2024 | 1 | 1 | 1 | 1 | 2 | 1 | 1 | 1 | 8 |
| Wei et al., 2023 | 1 | 1 | 1 | 1 | 1 | 1 | 1 | 0 | 7 |
