# Supplementary material for: Could the estrobolome have a role in endometriosis pathogenesis and infertility? A systematic review
Source: BMC Womens Health. 2025 Dec 18;26:43. doi: 10.1186/s12905-025-04195-z (PMC12821278; doi:10.1186/s12905-025-04195-z)
Supplement: Supplementary file 3 — Supplementary Material 3. [file 12905_2025_4195_MOESM3_ESM.docx]

**Additional file 3**. SYRCLE’s Risk of Bias Tool Evaluation for Animal Studies

| **Type of Bias** | **Domain** | **Alghetaa et al., 2023** | **Wei et al., 2023** |
| --- | --- | --- | --- |
| Selection bias | Sequence generation | Moderate | Moderate |
|  | Baseline characteristics | Moderate | Moderate |
|  | Allocation concealment | Unclear | Unclear |
| Performance Bias | Random housing | Unclear | Unclear |
|  | Blinding | Unclear | Unclear |
| Detection bias | Random outcome assessment | Moderate | Moderate |
|  | Blinding | Moderate | Moderate |
| Attrition bias | Incomplete outcome data | Low | Low |
| Reporting bias | Selective outcome reporting | Low | Moderate |
| Other | Other sources of bias | Moderate | Moderate |
